# Frightening Complex Visual Hallucinations in an Elderly Patient with Ophthalmological Pathology and Vascular Dementia

**DOI:** 10.1155/2020/8851761

**Published:** 2020-12-24

**Authors:** Francesca Hill, Matthew Spurr, Joseph Stratford

**Affiliations:** ^1^Gloucestershire Health and Care Trust, UK; ^2^University Hospitals Bristol NHS Trust, UK

## Abstract

A lady in her 90s was referred to the Later Life Team (LLT) in a rural area of the United Kingdom with complex visual hallucinations (VH). She had significant ophthalmological pathology, including cataracts, a branch retinal vein occlusion, and vitreous haemorrhage. The hallucinations included seeing monkeys ripping the heads off of her cats and lions prowling the garden. The patient was distressed by the hallucinations and believed them to be real events. Her management involved low dose olanzapine and requesting that her ophthalmological surgery be expedited. The surgery resulted in a significant reduction in VH. A diagnosis of vascular dementia went on to be made following cognitive testing and imaging. The cognitive impairment may have contributed to the patient's inability to identify her experiences as hallucinations and thus render her without insight. A review of the computed tomography (CT) scans performed prior to the patient's presentation to our service confirmed significant vascular pathology including small vessel disease and lacunar infarcts. Cognitive testing confirmed a cognitive impairment which had gone unnoticed by her family. This case leads to an interesting discussion regarding diagnosis in complex VH in cases of significant ophthalmological pathology but a lack of insight. Various authors have proposed theories to explain VH; cortical release and the Perception and Attention Deficit (PAD) model are explored as possible explanations for the experiences of this patient.

## 1. Introduction

Visual hallucinations (VH) in the elderly can be caused by a diverse range of conditions [1]. These range from neurodegenerative conditions (particularly Lewy Body dementia as well as other forms of dementia), Charles Bonnet Syndrome (CBS), delirium, and psychiatric conditions including schizophrenia. They can also be part of normal human experience, especially when in the transition to and from sleep [1].

VH in the visually impaired often prompt the clinician to consider a diagnosis of CBS, especially when the hallucinations are of a complex nature. However, there are diagnostic criteria which must be consulted before such a diagnosis can be made. These criteria generally set out the need for the patient to be cognitively intact and to retain at least partial insight; furthermore, some authors have stated that the coexistence of another mental illness which could explain the VH should exclude a diagnosis of CBS [2–4]. The need for the retention of insight into the hallucinatory nature of the experience was a stance initially laid out by Damas-Mora et al. in 1982, which was softened slightly by Gold and Rabins in 1989 when their criteria laid out that the insight need only be partial [2, 3]. The necessity for partial insight was again stated by Teunisse et al. in 1996 [4], when they updated Gold and Rabins' “checklist” (see Table 1). There has previously been a lack of inclusion in recognised diagnostic manuals such as the International Classification of Diseases version 10 (ICD-10) and the Diagnostic and Statistical Manual of Mental Disorders (DSM-V) of CBS [5, 6]; we have therefore relied on publications in medical journals to inform our practice. The ICD-11 is set to include CBS (which it terms “visual release hallucinations”); it is worth noting that there will be no criterion for retained insight [7]. The ICD-11 does state that “hallucinations are exclusively visual, usually temporary, and unrelated to mental or behavioural disorders” [7].

When insight is found to be lacking, a diagnosis of dementia should be considered. Dementia, including Alzheimer's disease, commonly presents with VH. In a literature review published in 2003, Bassiony and Lyketsos found that 19% of patients with Alzheimer's presented with VH. This is a greater proportion compared to Alzheimer's patients experiencing auditory hallucinations (12% of study participants) [8]. Complex visual hallucinations are also considered a key component of dementia with Lewy Bodies (DLB) and are estimated to occur in roughly 25% of patients with Parkinson's disease [9]. There has been comparatively less research into VH in the context of vascular dementia, and estimates of the prevalence of VH in the condition vary widely [10].

## 2. Case Presentation

Mrs. A, an elderly lady in her early 90s, presented to the Later Life Team (LLT) in a rural area in the United Kingdom with a range of hallucinations which she found immensely distressing. She lived with her daughter, granddaughter, and their respective husbands on a dairy farm. Her family was supportive and assisted her with her activities of daily living. Mrs. A experienced distressing VH that included monkeys ripping the heads off her cats and lions prowling in the garden and sleeping on the roof. She lacked insight, believing that the animals had escaped from her neighbours' house. There was no discernible relationship between the time of day and severity. Mrs. A engaged in risky behaviours, which included her trying to take control of the car whilst her daughter was driving in an attempt to avoid a VH of an unknown nature and wandering from the house, requiring her to be brought back by the police. The hallucinations had been present for over a year. It appeared that the trigger for Mrs. A's family seeking medical attention has been the increasing level of distress shown by Mrs. A in relation to the hallucinations and also the risky behaviour evident. Mrs. A displayed some unusual behaviours including referring to her granddaughter (a woman of roughly 40 years of age) as “Dorothy Powers,” saying that she is a very frail elderly lady. There were occasions where Mrs. A had appeared to be experiencing auditory hallucinations; this included reported hearing gramophone music playing in the distance.

At the time of the initial review with the psychiatric Later Life Team (LLT), Mrs. A was awaiting ophthalmological surgery for several pathologies. This included cataracts, a branch retinal vein occlusion, and vitreous haemorrhage. Her medical history included a suspected transient ischaemic attack (TIA) which had resulted in a loss of consciousness around two years prior to the review by the LLT. Other comorbidities included essential hypertension, diverticulitis, and vitamin D deficiency (for which she was receiving supplementation). Regarding her psychiatric history, she had seen her GP for anxiety and had been treated with flurazepam 15 mg ON for over 22 years. There had been no changes to this medication in the time preceding her experiencing hallucinations or prior to her being seen by the LLT. Other medications included vitamin D supplementation, dosulepin, losartan, and ranitidine. There was no significant family history to note.

The initial management involved contacting the ophthalmology department and explaining the impact that the visual impairment was having on her mental state and requesting that they consider expediting her surgery. Olanzapine 2.5 mg nocte was commenced; the family reported that this initially worsened Mrs. A's presentation which they described as “manic.” This settled after 24 hours, and Mrs. A then appeared less frightened of the hallucinations. When the olanzapine was increased to 5 mg ON, there was evidence of sedation, which led to it being decreased again to 2.5 mg ON which unfortunately resulted in a subsequent increase in distress. There was a discussion about reducing the flurazepam; however, it was felt that due to the duration of her treatment, it was unlikely to be contributing to the presentation. Furthermore, tolerance meant that it would be unlikely to be contributing to and worsening olanzapine-associated sedation and that attempting a dose reduction at this time may in fact worsen the patient's presentation.

The ophthalmological procedure took place five months after Mrs. A was first reviewed by the LLT. The surgery involved a combination of three procedures: a pars plana vitrectomy, phacoemulsification, and retinopexy. The surgery was successful, and the family reported a “marked decrease” in VH. When this was explored with Mrs. A, she stated that the monkeys had “gone next door.” Her belief that the monkeys had been present in the garden remained intact.

As a differential diagnosis would be a dementia process, an ACE-III was completed. The tester felt that Mrs. A's vision was good enough to allow her to engage in this process, and this was some time after the surgery when there had been an improvement in her vision. She scored 61/100. Up until this time, formal cognitive testing had not been completed. Mrs. A's family had reported that they had no concerns regarding her cognition; although it was noted that as she had several family members supporting her, this may have facilitated a cognitive impairment going unnoticed as she did not have to complete tasks such as cooking or shopping. Furthermore, Mrs. A's misidentification of her granddaughter and wandering are evidence that dementia had been present.

**Figure 1:**
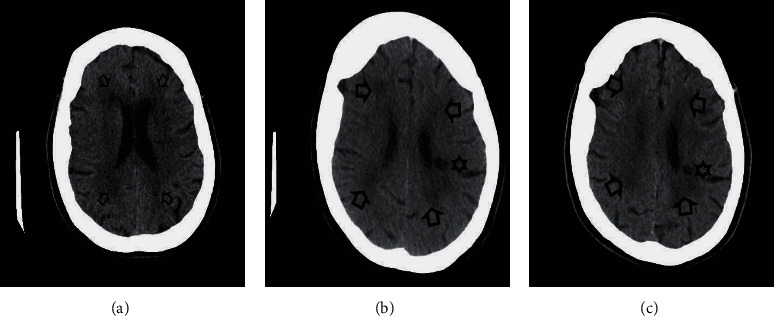
Axial noncontrast computed tomography of the head from 2017 to 2019 ((a) May 2017, (b) August 2018, and (c) October 2019). Key: arrow = hypoattenuation in the deep white matter is in keeping with moderate chronic small vessel ischaemic disease. Appearances are stable from 2017 to 2019. Star = chronic lacunar infarct in the left parietal lobe (first visualised in 2018).

Three computed tomography (CT) scans of her head had been performed—two prior to the presentation to the LLT, one after (see Figure 1). These scans were performed due to a fall in the bathtub (Figure 1(a)), a presentation to the Accident and Emergency Department due to confusion (Figure 1(b)), and due to a period of unresponsiveness (Figure 1(c)). The referral to the LLT took place in April 2019. The imaging reveals severe small vessel disease which has been stable since initial imaging in 2017. Two lacunar infarcts were seen: one in the lentiform nucleus/anterior limb of the internal capsule and one in the left parietal lobe. A radiologist has confirmed that the infarcts are not located within the visual pathway. Other investigations have included a blood test, which revealed poor renal function with a baseline eGFR of 35-45. HBA1c, thyroid function, and haematinics have consistently been within range.

Due to the CT head appearances alongside demonstrated cognitive impairment, a diagnosis of dementia was made; the dementia was deemed to be likely vascular in nature due to the appearances on imaging. Whilst an expected minimal response to medication was explained to the patient and her family, they remained keen to trial medication and so memantine at a low dose (5 mg OD) on account of renal impairment was trialled. This was stopped by Mrs. A's daughter as she felt it was causing sedation.

There was no evidence of motor involvement, with Mrs. A's family describing her as “sprightly.” She was still fairly active, which led to some issues due to her wandering from the property. No tremor was detectable, and there was no micrographia. Further investigations to rule out Lewy Body dementia were therefore not felt to be indicated.

## 3. Discussion

Due to the presence of “fantastical” complex VH alongside significant ophthalmological pathology, the seemingly obvious diagnosis at the time was CBS. This was especially in the context of the improvement in VH following ophthalmological surgery, which the authors initially took as evidence for CBS. However, there are a number of factors which must be considered which are evidence that a diagnosis of CBS cannot be made in the case of Mrs. A.

We will first address the initial view of the team that the reduction in VH following surgery was a testament to the diagnosis being CBS. As with CBS, visual acuity in dementia plays a key role in the development of VH. Furthermore, correction of visual deficits can lead to resolution of VH in dementia patients. In a study of 50 patients with suspected Alzheimer's, 20 of whom experienced VH, Chapman et al. found that impaired visual acuity was significantly associated with VH [11]. Particularly relevant to Mrs. A was the finding that correction of visual deficit (using corrective lenses) had a beneficial impact on VH [11]. Cataracts were also found to be significantly more common in the hallucinators compared to the nonhallucinators [11]. It would appear that the contribution of visual deficit to the development of VH is not exclusive to CBS.

Lack of exclusivity of poor vision resulting in VH to CBS is not the only evidence against CBS as a diagnosis here. Consulting the Teunisse checklist (1999) reveals that the case for Mrs. A having a diagnosis of CBS is weak. This is due to the lack of insight into the hallucinatory nature of her experiences, the absence of hallucinations in other modalities (note the presence of auditory hallucinations), and the absence of delusions (which she displayed through her bizarre and unshakeable belief that her granddaughter was an elderly lady named Dorothy Powers).

The presence of vascular dementia was further evidence against the diagnosis of CBS given that vascular dementia itself is associated with VH [10]. The diagnosis had not been made at the time of Mrs. A's review with the LLT, although, from the examination of her imaging, it is clear that the vascular pathology was present prior to the LLT's initial assessment (see Figure 1). In hindsight, it is likely that Mrs. A's unusual behaviour towards her granddaughter (calling her Dorothy Powers and referring to her as an old lady) was a result of cognitive decline secondary to vascular dementia. The wandering is also likely to have been secondary to cognitive decline. It is suspected that cognitive deficit impaired Mrs. A's ability to identify her experiences as hallucinations. Whilst some authors have argued that patients with dementia or absent insight could be diagnosed with CBS [12, 13], Teunisse himself has rebutted such claims [14]. In a systematic review completed in 2014, Russell and Burns did not identify a single study which appropriately used accepted diagnostic criteria and that were adequately powered to be able to draw adequate conclusions regarding the relationship between cognition and CBS, and whether CBS could be a precursor to dementia [15]. The same authors published a prospective cohort study in 2017 which explored the difference in conversion to dementia in CBS and non-CBS (but visually impaired) groups; they were unable to detect a difference between groups and therefore were unable to find evidence that CBS is a precursor to dementia, although the small sample size should be noted [16].

The pathophysiology of VH is another topic of some debate. It has long been postulated that depriving the visual cortex of stimulus may result in hyperactivity, which underpins the VH in reduced visual acuity. Functional magnetic resonance imaging studies have shown tonic activity in the visual pathways, occurring both during the hallucination and also when the person is not experiencing VH [1, 17, 18]. Further evidence for this theory is the production of hallucinations (including visual) when sensory deprivation is intentionally produced in a laboratory environment. Mohan and Vanneste term this “phantom percept,” explaining that the abnormal perception (or VH in this case) is the result of the brain's maladaptive attempt to compensate for its lack of stimulus. They reference phantom pain as experienced by amputees and musical hallucinosis in the deaf as other examples [19]. Other models for VH include cortical irritation, cortical hyperexcitability, and dream intrusion [11]. However, there are limitations to these models which are perhaps addressed by the Perception and Attention Deficit (PAD) model as proposed by Collerton et al. [20].

Collerton and his colleagues propose that a combination of deficits in object-based attention, object-based perception, and intact sensory representation is key for the development of VH [1, 20]. They describe the insertion of a hallucinatory “proto-object” in the context of preserved scene perception—in the case of Mrs. A, this represented tropical animals being seen in her garden. Interestingly, Collerton suggests that certain scenes can invoke VH which can explain why people report repeatedly experiencing VH in the same context each time—again, this is reminiscent of Mrs. A whose VH primarily occurred in the context of the garden scenery. The model was initially developed to account for the VH in Lewy Body dementia, although it can be expanded to account for VH in both pathological and nonpathological states including degenerative neurological conditions, and also hypnagogic and hypnopompic VH [20].

The case of Mrs. A is a reminder that VH in a visually impaired person is not necessarily a result of CBS and that a diagnosis of dementia should be considered. There is a need for high quality randomised controlled trials aimed at investigating the impact of correcting visual deficits in patients with dementia and VH. This could lead to an improvement in the quality of life of the person given the distress that a person may experience as a result of the VH, as seen with Mrs. A.

## Figures and Tables

**Table 1:** The criteria laid out by Teunisse et al. (1996).

At least one complex visual hallucination in the past four weeks
A period between the first and last hallucination exceeding four weeks
Full or partial retention of insight into the unreal nature of the hallucinations
Absence of hallucinations in other sensory modalities
Absence of delusions

## Data Availability

No data were used to support this study.
